# Surveillance of Extended-Spectrum β-Lactamase-, Cephalosporinase- and Carbapenemase-Producing Gram-Negative Bacteria in Raw Milk Filters and Healthy Dairy Cattle in Three Farms in *Île-de-France*, France

**DOI:** 10.3389/fvets.2021.633598

**Published:** 2021-02-10

**Authors:** Vincent Plassard, Philippe Gisbert, Sophie A. Granier, Yves Millemann

**Affiliations:** ^1^Ecole Nationale Vétérinaire d'Alfort, Maisons-Alfort, France; ^2^Ceva Santé Animale, Libourne, France; ^3^Agence Nationale de Sécurité Sanitaire de l'Alimentation, de l'Environnement et du Travail, Fougères, France; ^4^Laboratoire de Sécurité des Aliments de l'ANSES, Maisons-Alfort, France

**Keywords:** antimicrobial resistance, carbapenemase, extended-spectrum beta-lactamase, multi-drug resistance, dairy cattle, gram-negative

## Abstract

The aim of this work was to test a surveillance protocol able to detect extended-spectrum β-lactamase (ESBL)-, cephalosporinase (AmpC)- and carbapenemase (CP)-producing gram-negative bacteria in three conveniently chosen dairy farms with known prior occurrences of ESBL- and CP-producing strains. The protocol was applied monthly for a year. At each visit, 10 healthy lactating dairy cows were rectally swabbed, and raw milk filters (RMFs) were sampled in two of the three farms. Bacterial isolation was based on a first screening step with MacConkey agar supplemented with 1 mg/L cefotaxime and commercial carbapenem-supplemented media. We failed to detect CP-producing strains but showed that ESBL-*Escherichia* strains, found in one farm only (13 strains), were closely associated with multi-drug resistance (12 out of 13). The limited number of conveniently selected farms and the fact that RMFs could not be retrieved from one of them limit the validity of our findings. Still, our results illustrate that ESBL-status changes monthly based on fecal swabs and negative herds should be qualified as “unsuspected” as proposed by previous authors. Although surveillance of farm statuses based on RMF analysis could theoretically allow for a better sensitivity than individual swabs, we failed to illustrate it as both farms where RMFs could be retrieved were constantly negative. Determination of CP herd-level status based on RMFs and our surveillance protocol was hindered by the presence of intrinsically resistant bacteria or strains cumulating multiple non-CP resistance mechanisms which means our protocol is not specific enough for routine monitoring of CP in dairy farms.

## Introduction

Extended-spectrum β-lactamase (ESBL)-, cephalosporinase (AmpC)- and carbapenemase (CP)-producing gram-negative (GN) bacteria have been described in numerous settings of the ecosphere worldwide (1), including cattle (2, 3). An increasing amount of evidence of gut colonization in cattle with ESBL-/AmpC- (4–7) and CP-producing GN bacteria (8, 9) is accruing, especially in commensal *Enterobacteriaceae*, among healthy beef cattle (10, 11) and healthy dairy cattle (8, 12–16). ESBL-/AmpC-/CP-producing GN bacteria have also been reported in raw milk (17, 18). In the One Health approach the presence of these bacteria in livestock such as cattle creates a general concern of the scientific community who sees the animal sector as a reservoir for selection and amplification of antimicrobial resistance genes by the use of antibiotics before they eventually spread back to humans (1). Data gaps still exist which prevent a full quantitative risk analysis of possible transmission routes through livestock to humans, e.g., levels of ESBL-producing *Escherichia coli* in the bulk tank milk (BTM) on farm (19) and CP-reports using systematic screening with carbapenem-supplemented selective media instead of side investigations within other studies (1).

Indeed, only a few studies actually address the presence of nonpathogenic or commensal antimicrobial resistant bacteria specifically in BTM as a main research project (20–22), most of them being focused on the detection and antimicrobial profiling of zoonotic foodborne pathogens [e.g., (23–26)]. Instead of BTM, or concomitantly with BTM, some authors advocate the analysis of raw milk-filters (RMFs) as a way to monitor the presence of zoonotic foodborne pathogens in a dairy herd (27) or in raw milk (23, 28–31). In-line RMFs are an essential part of milking systems, trapping particles of organic material and foreign objects, but they are not designed to ward off bacteria. Residues withheld in the RMFs are considered as real-time indicators of the fecal shedding of zoonotic pathogens from a milking herd and its environment (27, 30) or indicators of the presence of resistant commensal *E. coli* in dairy herds (32–34). Overall, these studies illustrate the usefulness of RMF analysis as a way to monitor the presence of both zoonotic foodborne pathogens and commensal antimicrobial resistant bacteria in dairy herds.

Therefore, we conducted a longitudinal study focused on monitoring the presence of ESBL-/AmpC-/CP-producing GN bacteria in three dairy farms by using RMF as a proxy for dairy cows fecal shedding of resistant GN enteric bacteria. RMF analysis was associated with concomitant fecal samplings of healthy lactating dairy cattle in order to assess the baseline of resistant GN enteric bacteria in dairy cows in each farm. The objective of this study was to devise and test this surveillance protocol which would allow repeated point-of-care diagnostics able to detect the presence of ESBL-/AmpC-/CP-producing GN bacteria at the dairy farm level.

## Materials and Methods

### Herd Selection and Farm Visits

Three suburban conventional dairy farms (i.e., non-organic) were selected, all located in the same department at the South-West of Paris. During the year 2018, the mean number of lactating cows was 393 in Farm A, 168 in Farm B and 150 in Farm C. This choice was a convenient one as these farms are regularly visited for routine reproductive follow-ups by members of our teaching team. Besides, these farms had already been included in previous studies conducted by members of our research team which demonstrated the intestinal colonization of cows in Farm A by CP-producing *Acinetobacter* in 2010 (9), and the intestinal colonization of cows in Farms A and B by ESBL-producing *E. coli* in 2015 (35). The study lasted 1 year, from January 2018 to December 2018; samplings were scheduled once a month in each farm.

### Cow Selection

At each sampling session, a convenient number of ten cows were randomly selected among the 40–90 cows presented for a routine reproductive follow-up using random number generator from Excel (Microsoft Corporation, version 2013). Reasons for their inclusion in routine reproductive follow-up were uterine involution control, pregnancy diagnosis at 1, 3, or 7 months, and anestrus. To be included in the study, a cow needed to be clinically healthy after clinical examination by the first author, and its milk had to be currently collected. Characteristics of sample populations in each farm are presented in Supplementary Table 1.

### Samples Collection

Rectal swabs were performed using a swab with Amies agar (Copan, Brescia, Italy). RMFs were collected at the end of the morning milking session, before the cleaning and rinse procedures, and placed in a sterile plastic stomacher bag (Fisher Scientific, Illkirch, France) then sealed. Only Farm B and Farm C used milk filters, Farm A used a percolation system to trap debris which unfortunately prevented the addition of a filter. This information wasn't mentioned by the farm manager at enrollment. All samples were stored on ice and delivered to the laboratory within 4 h from collection. Once arrived in the laboratory, the samples were immediately processed.

### Laboratory Screening Procedures

Swabs were placed aseptically in 9 mL buffered peptone water (BPW) (bioMérieux, Marcy l'Etoile, France). RMFs were unsealed, kept in the stomacher bag, soaked with 225 mL of BPW (bioMérieux, Marcy l'Etoile, France) and sealed again. The stomacher bag was then shaken manually. All samples were incubated at 37 ± 2°C for 4–6 h for pre-enrichment. Antimicrobial resistant isolates were sought with three different selective media: ChromID™ CARBA (bioMérieux, Marcy l'Etoile, France), ChromID™ OXA-48 (bioMérieux, Marcy l'Etoile, France) and a non-commercial selective MacConkey (McC) agar plate (Condalab, Madrid, Spain) supplemented with 1 mg/L cefotaxime (COX) (Sigma-Aldrich, Saint-Louis, MO, USA). Quality of the latter media was controlled before each use with a negative strain *E. coli* ATCC25922 and a positive strain *E. coli* NCTC13353. Plates were inoculated with 10 μL of enriched solution, placed at 37 ± 2°C for 18–24 h for a first reading, and then replaced at 37 ± 2°C for another 18–24 h for a second reading. At the last reading, phenotypically different and isolated colonies were numbered and smeared on the corresponding media then incubated for another 24 h at 37 ± 2°C. Once purity was confirmed, a colony was taken and stored at −80°C using cryo-beads (bioMérieux, Marcy l'Etoile, France).

### Microbial Identification

Phenotypically resistant strains were identified to the genus- and species-level using matrix-assisted laser desorption/ionization time-of-flight mass spectrometry (MALDI-TOF MS). Isolates were processed on a Biotyper Microflex LT^®^ using Flex Control software (Bruker Daltonics, Champs-sur-Marne, France). The MALDI-TOF target plate was prepared using the extraction method given by the manufacturer. Species identification cut-off values were applied according to the manufacturer's instructions, according to which a score of ≥2 indicated a species-level identification, a score between ≥1.7 and <2 indicated a genus-level identification. Whatever the isolate, a score <1.7 was considered inconclusive. In that case, the sample was cultured again on a brain heart infusion agar at 37 ± 2°C for reprocessing the next day. Failure to obtain at least an identification to the genus-level prompted an exclusion from further analysis.

Non-GN isolates and intrinsically resistant GN isolates were excluded from further analysis. Briefly, *Pseudomonas* spp. (36), *Acinetobacter* spp. (36–38), *Achromobacter* spp. (39), *Wautersiella falsenii* (40), and *Empedobacter brevis* (41) were considered naturally resistant to COX; *Stenotrophomonas maltophilia* (36) and *Arcobacter butzleri* (42) were considered naturally resistant to carbapenems.

### Antimicrobial Susceptibility Testing

All GN isolates for which growth on McC-COX, ChromID™ CARBA or ChromID™ OXA-48 could not be attributed to intrinsic resistance to COX or carbapenems were submitted to antimicrobial susceptibility testing (AST) by disc diffusion with a panel of 15 antimicrobial agents (Supplementary Table 2) on Mueller-Hinton agar (bioMérieux, Marcy l'Etoile, France). The EUCAST standards were followed for inoculum standardization and incubation conditions (43). Quality control was performed using *E. coli* ATCC25922 (44). Antimicrobial disks (Biorad, Marnes-la-Coquette, France) were spaced by a standard distance of 30 mm from each other. Inhibition zone diameter (IZD) readings were made following EUCAST recommendations (45); results are available for all strains as supplemental material (Supplementary Table 3). When available, EUCAST epidemiological cut-off values (ECOFF) were applied to determine “microbiological” resistance (46), otherwise EUCAST clinical breakpoints were used (43) (Supplementary Table 2). Of note, neither the ECOFF nor the clinical breakpoint for tetracycline (TET) for *Enterobacteriaceae* being available in the EUCAST database, the 2018 CLSI clinical breakpoint was applied for this antimicrobial agent after verification that incubation methods were compatible (47). Antimicrobial resistance patterns for each strain constituted the basis for determining duplicates originating from the same samples, i.e., bacteria of the same genus and species isolated from the same matrix on the same media and on the same day. Duplicates were excluded from final data presentation.

This panel was a compromise intended to allow both detection of ESBL-/AmpC-/CP-phenotypes and assessment of multi-drug resistance (MDR) among *Enterobacteriaceae* and Pseudomonads which were the most frequently identified families. The panel allowed: (i) screening of *E. coli* strains stably overproducing AmpC β-lactamase by applying a breakpoint of <19 mm for cefoxitin (FOX) (43); (ii) detection of inducible-AmpC production among *Enterobacteriaceae* and *Pseudomonas* spp. by performing a Ceftazidime-Imipenem Antagonism Test (CIAT) (48); (iii) detection of ESBL-producing *Enterobacteriaceae* strains by performing three double-disk synergy tests [DDSTs; amoxicillin-clavulanic acid (AMC) next to COX, ceftazidime (CZD) and cefepime (FEP)] (49); (iv) detection of a subsample of potentially CP-producing strains among ChromID™ CARBA and ChromID™ OXA-48 isolates based on imipenem (IPM) EUCAST ECOFF when available, EUCAST clinical breakpoint otherwise. This subsample of IPM-resistant strains was subsequently tested for CP production by using a commercial RAPIDEC^®^ CARBA NP test (bioMérieux, Marcy l'Etoile, France); (v) definition of MDR status, i.e., “microbiological” resistance to three or more antimicrobial classes (46, 50).

The algorithm used for determining the isolates' ESBL-/AmpC-/CP-statuses is summarized in Supplementary Figure 1.

### Definition of ESBL/AmpC/CP Herd Status

Herds that tested positive for ESBL-/AmpC-/CP-producing GN bacteria in any number of cows or in RMFs were defined as positive. Following the terminology used by previous authors (12, 51), negative herds were defined as unsuspected.

## Results

### Farm Characteristics and Sample Descriptions

Farms A and B were visited 11 times during the year 2018, Farm C 12 times, with a mean number of 32 days between visits in Farms A and B, and 29 days in Farm C. Overall, 338 rectal swabs were performed, with 110 animals sampled in Farms A and B, and 118 in Farm C; 23 RMFs were sampled in Farms B and C, with, respectively, 11 and 12 RMFs for each farm. Two rectal swabs were excluded in Farm C at the December visit because of an identification error. The median cow characteristics at the day of sampling are presented in Supplementary Table 1. As sampled cows were randomly selected among cows presented for reproductive motives, some animals were sampled multiple times during the year 2018: 12, 20, and 28 cows were therefore sampled at least two times in Farms A, B, and C, respectively.

### Microbial Identification and Frequency of Genera and Species

In Farm A, 72 isolates were submitted to identification. Using MALDI-TOF, a total of 62 (86%) isolates from fecal swabs were identified at least to the genus-level. Non-GN organisms were predominantly isolated (41 isolates, 57%), especially *Candida* spp. which represented 29 of the 72 isolates (40%). The second most predominant genus in Farm A was *Escherichia* spp. which represented 13 out of the 72 isolates (18%). Within the 13 *Escherichia* spp., five were identified to the species-level as *E. coli*.

In Farm B, 20 isolates of fecal swab origin were submitted to identification and all of them were identified at least to the genus-level. Again, non-GN organisms were dominant (13 isolates, 65%), especially *Candida* spp. which represented 12 of the 20 isolates (60%). Ninety-one isolates of RMF origin were submitted to identification, and 71 (88%) were identified at least to the genus-level. Contrary to fecal swab isolates, non-GN organisms were scarcely isolated in RMF (two out of 81, 2%). The most predominant genus was by far *Pseudomonas* spp., which represented 57 isolates out of 81 (70%). Within this genus, 27 could be identified to the species-level, with *Pseudomonas aeruginosa* being the most frequent with 24 isolates. *Pseudomonas* spp. were isolated from RMFs at ten out of 11 visits, only December was spared.

In Farm C, 35 isolates of fecal swab origin were submitted to identification. A total of 29 (83%) of these were identified at least to the genus-level. Once again, non-GN organisms were predominant with 28 isolates (80%), including 26 *Candida* spp. (74%). One hundred and fourteen isolates of RMF origin were submitted to identification, among which 97 isolates were identified at least to the genus-level. Similarly as in Farm B and in opposition to fecal swabs, only one non-GN organism was isolated. Among the GN organisms of RMF origin, the most frequent genus was again *Pseudomonas* spp. (65 isolates, 57%); 29 isolates could be identified to the species-level with *Pseudomonas aeruginosa* being the most frequent one with 23 isolates. *Pseudomonas* spp. were isolated from RMFs at eight out of 12 visits, the months of January, September and November were spared.

Numbers and proportions of each genus and species are presented in Table 1.

**Table 1:** Number (percent) of each genera identified among isolates of fecal swab or raw milk filter origin within each farm over the year 2018.

**Isolates of fecal swab origin**										
***Genus***	**Farm A no. (%**, ***n*** **=** **72)**			**Farm B no. (%**, ***n*** **=** **20)**			**Farm C no. (%**, ***n*** **=** **35)**			**Total (%**, ***n*** **=** **127)**
	**McC** **+** **COX**	**CARBA OXA-48**	**Total**	**McC** **+** **COX**	**CARBA OXA-48**	**Total**	**McC** **+** **COX**	**CARBA OXA-48**	**Total**	
**Inconclusive**^a^	**2 (3%)**	**8 (11%)**	**10 (14%)**	**0 (0%)**	**0 (0%)**	**0 (0%)**	**2 (6%)**	**4 (12%)**	**6 (17%)**	**16 (13%)**
**Non-gram-negative**^b^	**13 (18%)**	**28 (39%)**	**41 (57%)**	**3 (15%)**	**10 (50%)**	**13 (65%)**	**0 (0%)**	**28 (80%)**	**28 (80%)**	**82 (65%)**
*Candida* spp.^b^	8 (11%)	21 (29%)	29 (40%)	2 (10%)	10 (50%)	12 (60%)	0 (0%)	26 (74%)	26 (74%)	67 (53%)
**Gram-negative**	**16 (22%)**	**5 (7%)**	**21 (29%)**	**4 (20%)**	**3 (15%)**	**7 (35%)**	**0 (0%)**	**1 (3%)**	**1 (3%)**	**29 (23%)**
*Acinetobacter* spp.^b^	0 (0%)	0 (0%)	0 (0%)	2 (10%)	0 (0%)	2 (10%)	0 (0%)	0 (0%)	0 (0%)	2 (2%)
*Bordetella* spp.^b^	0 (0%)	0 (0%)	0 (0%)	1 (5%)	0 (0%)	1 (5%)	0 (0%)	0 (0%)	0 (0%)	1 (<1%)
*Enterobacter* spp.^b^	0 (0%)	2 (3%)	2 (3%)	0 (0%)	0 (0%)	0 (0%)	0 (0%)	0 (0%)	0 (0%)	2 (2%)
*Ochrobactrum* spp.^b^	0 (0%)	0 (0%)	0 (0%)	0 (0%)	2 (10%)	2 (10%)	0 (0%)	0 (0%)	0 (0%)	2 (2%)
*Escherichia* spp.^(b)^	13 (18%)	0 (0%)	13 (18%)	0 (0%)	0 (0%)	0 (0%)	0 (0%)	1 (3%)	1 (3%)	14 (11%)
Including *E. coli*^c^	*5 (7%)*	*0 (0%)*	*5 (7%)*	*0 (0%)*	*0 (0%)*	*0 (0%)*	*0 (0%)*	*1 (3%)*	*1 (3%)*	*6 (5%)*
*Pseudomonas* spp.^b^	3 (4%)	3 (4%)	6 (8%)	1 (5%)	1 (5%)	2 (10%)	0 (0%)	0 (0%)	0 (0%)	8 (6%)
Including *P. aeruginosa*^c^	*1 (1%)*	*0 (0%)*	*0 (0%)*	*0 (0%)*	*1 (5%)*	*1 (5%)*	*0 (0%)*	*0 (0%)*	*0 (0%)*	*3 (2%)*
Total	**31 (43%)**	**41 (57%)**	**72 (100%)**	**7 (35%)**	**13 (65%)**	**20 (100%)**	**2 (6%)**	**33 (94%)**	**35 (100%)**	**127 (100%)**
**Isolates of raw milk filter origin**										
***Genus***				**Farm B no. (%**, ***n*** **=** **81)**			**Farm C no. (%**, ***n*** **=** **114)**			**Total (%**, ***n*** **=** **195)**
				**McC** **+** **COX**	**CARBA OXA-48**	**Total**	**McC** **+** **COX**	**CARBA OXA-48**	**Total**	
**Inconclusive**^a^				**5 (6%)**	**5 (6%)**	**10 (12%)**	**4 (4%)**	**13 (11%)**	**17 (15%)**	**27 (14%)**
**Non-gram-negative**^b^				**0 (0%)**	**2 (2%)**	**2 (2%)**	**0 (0%)**	**1 (1%)**	**1 (1%)**	**3 (2%)**
**Gram-negative**				**25 (31%)**	**44 (55%)**	**69 (85%)**	**34 (30%)**	**62 (54%)**	**96 (84%)**	**165 (85%)**
*Achromobacter* spp.^b^				0 (0%)	0 (0%)	0 (0%)	1 (1%)	0 (0%)	A (1%)	1 (<1%)
*Acinetobacter* spp.^b^				3 (4%)	1 (1%)	4 (5%)	5 (4%)	0 (0%)	5 (4%)	9 (5%)
*Aeromonas* spp.^b^				0 (0%)	1 (1%)	1 (1%)	0 (0%)	6 (5%)	6 (5%)	7 (4%)
*Arcobacter* spp.^b^				0 (0%)	0 (0%)	0 (0%)	0 (0%)	2 (2%)	2 (2%)	2 (1%)
*Empedobacter* spp.^b^				0 (0%)	0 (0%)	0 (0%)	1 (1%)	0 (0%)	1 (1%)	1 (<1%)
*Myroides* spp.^(b)^				0 (0%)	0 (0%)	0 (0%)	0 (0%)	3 (3%)	3 (3%)	3 (2%)
*Ochrobactrum* spp.^b^				0 (0%)	1 (1%)	1 (1%)	0 (0%)	1 (1%)	1 (1%)	2 (1%)
*Stenotrophomonas* spp.^b^				0 (0%)	5 (6%)	5 (6%)	0 (0%)	9 (8%)	9 (8%)	14 (7%)
*Wautersiella* spp.^b^				1 (1%)	0 (0%)	1 (1%)	3 (3%)	0 (0%)	3 (3%)	4 (2%)
*Pseudomonas* spp.^b^				21 (26%)	36 (44%)	57 (70%)	24 (21%)	41 (36%)	65 (57%)	122 (63%)
Including *P. aeruginosa*^c^				*12 (15%)*	*12 (15%)*	*24 (30%)*	*14 (12%)*	*9 (8%)*	*23 (20%)*	*47 (24%)*
Total				**30 (37%)**	**51 (63%)**	**81 (100%)**	**38 (33%)**	**66 (67%)**	**114 (100%)**	**195 (100%)**

*McC + COX, MacConkey + Cefotaxime; CARBA, ChromID ™ CARBA; OXA-48, ChromID™ OXA-48*.

a *Isolates for which MALDI-TOF score was <1.7*.

b *Isolates for which MALDI-TOF score was >1.7 and <2*.

c *Isolates for which MALDI-TOF score was >2*.

### Phenotypic Screening

In Farm A, out of the 62 isolates of fecal origin identified at least to the genus level, 18 were submitted to AST (Table 2): eight *Escherichia* spp. and five *E. coli* isolated on McC + COX; one *Pseudomonas* spp., two *Pseudomonas fragi* and two *Enterobacter* spp. isolated on ChromID™ CARBA and ChromID™ OXA-48. All the *Escherichia* isolates were confirmed resistant to COX, ESBL-producers according to the DDSTs, non-AmpC-producers according to FOX screening, and MDR for 12 out of the 13 strains (92%) (Table 3). The most frequent resistance profile patterns, five out of 13 isolates (38.5%), associated resistance to ampicillin (AMP), COX, FEP, TET, and trimethoprim-sulfamethoxazole (SXT) (Table 4). ESBL-producing *Escherichia* strains were isolated in March (one out of 13), May (one out of 13), June (seven out of 13), July (three out of 13) and December (one out of 13). None of the *Pseudomonas* and *Enterobacter* were resistant to IPM, in fact they were all pansusceptible to the panel of antibiotics except for intrinsic resistances. The CIAT was positive for both the *Enterobacter* strains. Based on our phenotypic surveillance protocol, Farm A was classified CP-negative for the year 2018, and ESBL-positive at five sampling periods out of 11 (March, May, June, July, December). The proportions of positive cows harboring ESBL-producing enteric GN bacteria were highest in June and July, at, respectively, 70% (seven out of ten) and 30% (three out of ten); they were preceded by low-levels of positivity in March and May, both at 10% (one cow out of ten) followed by a 10% positivity in December and unsuspected cows in-between. Interestingly, among the six ESBL-producing *Escherichia coli* in June, July and December, five of them were lactose-negative on McC + COX after 48 h, and only the one strain isolated in December was lactose-positive.

**Table 2:** Occurrence of resistance to cefotaxime and imipenem in gram-negative bacteria isolated, respectively, on McC + COX, and ChromID™ CARBA and ChromID™ OXA-48, in each farm in 2018.

		**Strains isolated on McC** **+** **COX**				**Strains isolated on ChromID**™ **CARBA and OXA-48**			
		**Total no. of isolates^a^**	**No. of isolates identified^b^ (%)**	**No. of isolates submitted to AST^c^ (%)**	**No. of isolates COX-resistant^d^^,^^e^ (%)**	**Total no. of isolates^a^**	**No. of isolates identified^b^ (%)**	**No. of isolates submitted to AST^c^ (%)**	**No. of isolates IPM-resistant^d^^,^^e^ (%)**
Fecal swabs	Farm A	31	29 (94%)	13 (42%)	13 (42%)	44	33 (75%)	5 (11%)	0 (0%)
	Farm B	7	7 (100%)	0 (0%)	0 (0%)	14	13 (93%)	1 (7%)	0 (0%)
	Farm C	2	0 (0%)	0 (0%)	0 (0%)	34	29 (85%)	1 (3%)	0 (0%)
	Total	**40**	**36 (90%)**	**13 (36%)**	**13 (36%)**	**92**	**75 (82%)**	**7 (8%)**	**0 (0%)**
Raw milk filters	Farm B	30	25 (83%)	0 (0%)	0 (0%)	53	46 (87%)	38 (72%)	4 (8%)
	Farm C	41	37 (90%)	0 (0%)	0 (0%)	76	60 (79%)	46 (61%)	2 (3%)
	Total	**71**	**62 (87%)**	**0 (0%)**	**0 (0%)**	**129**	**106 (82%)**	**84 (65%)**	**6 (5%)**

*Percentages are based on the total number of isolates in respective farms*.

a *Number of strains phenotypically different isolated on selective media*.

b *Number of isolates identified at least to the genus-level with MALDI-TOF*.

c *Number of isolates submitted to AST after exclusion of isolates intrinsically resistant to COX or carbapenems based on genus and/or species*.

d *“Microbiological” resistance based on EUCAST ECOFF when available; on EUCAST clinical breakpoints 2020 if not*.

e *Final number after exclusion of duplicate strains based on antimicrobial susceptibility profiles*.

**Table 3:** Summary of presumptive ESBL-/AmpC-/CP-producing *Escherichia* spp. and *Pseudomonas* spp. collected within the monitoring in 2018.

**Matrix**	**Farm**	**Selective media**	**No. of presumptive ESBL producers (%)^a^**	**No. of presumptive AmpC producers (%)**	**No. of presumptive CP producers (%)^d^**	**No. of MDR isolates (%)^e^**
***Escherichia*** **spp**.						
Fecal swabs	Farm A (*n* = 13)	McC + COX	13 (100%)	0 (0%)^b^	ND	12 (92%)
***Pseudomonas*** **spp**.						
Raw milk filters	Farm B (*n* = 4)	CARBA	0 (0%)	4 (100%)^c^	0 (0%)	0 (0%)
	Farm C (*n* = 2)	CARBA	0 (0%)	2 (100%)^c^	0 (0%)	0 (0%)

*n = isolates showing “microbiological” resistance to cefotaxime and isolated on McC + COX, or isolates showing “microbiological” resistance to imipenem and isolated on ChromID™ CARBA or OXA-48 were considered (see Materials and Methods); ND, non-determined*.

a *Isolates showing clavulanate synergy with cefotaxime, ceftazidime, cefepime or all compounds, suggesting the presence of an ESBL (indepedently of the presence of other mechanisms)*.

b *Escherichia isolates showing “microbiological” resistance to cefoxitin (see Materials and Methods)*.

c *Pseudomonas isolates showing ceftazidime inhibition with imipenem (see Materials and Methods)*.

d *Based on a Rapidec© CARBA NP test (see Materials and Methods)*.

e *Based on the classification by Magiorakos et al. (50)*.

**Table 4:** Resistance patterns of *Escherichia* strains isolated from dairy cows' rectal swabs in Farm A (*n* = 13) on the basis of inhibition zone diameter breakpoints^a^.

**Resistance profile**	**No. of isolates with profile**	**% of isolates with profile**	**MDR status^b^**
AMP COX	1	7.7%	Non-MDR
AMP COX FEP TET	1	7.7%	MDR
AMP COX SXT TET	2	15.4%	MDR
AMP COX FEP TET SXT	5	38.5%	MDR
AMP COX FEP TET SXT LVX	1	7.7%	MDR
AMP COX FEP SXT CIP LVX	1	7.7%	MDR
AMP COX FEP TET SXT CIP LVX	2	15.4%	MDR

a *“Microbiological” resistance based on EUCAST ECOFF when available; EUCAST clinical breakpoint 2020 if not; CLSI 2018 if none available*.

b *Magiorakos et al. (50)*.

*AMP, ampicillin; FEP, cefepime; COX, cefotaxime; TET, tetracycline; SXT, trimethoprim-sulfamethoxazole; CIP, ciprofloxacin; LVX, levofloxacin*.

In Farm B, out of the 20 isolates of fecal origin identified to the genus-level, only one was submitted to AST (Table 2): a *P. aeruginosa* isolated on ChromID™ CARBA in June, which was susceptible to IPM. The CIAT was positive, meaning the strain was an inducible AmpC-producer. Out of the 71 isolates of RMF origin identified to the genus-level, 38 were submitted to AST (Table 2): 21 *Pseudomonas* spp., 12 *P. aeruginosa*, two *Pseudomonas koreensis*, one *Pseudomonas mendocina*, one *Acinetobacter proteolyticus*, and one *Aeromonas hydrophila*. All of these strains were isolated on ChromID™ CARBA or ChromID™ OXA-48 media. Three *Pseudomonas* spp. and 11 *Pseudomonas aeruginosa* were resistant to IPM, none of them were MDR. All the other isolates, including *Aeromonas hydrophila* and *A. proteolyticus*, were pansusceptible. All the *Pseudomonas* spp. and *P. aeruginosa* IPM-resistant strains and one *P. aeruginosa* IPM-susceptible strain were inducible AmpC-producers based on the CIAT. The subsample of 14 IPM-resistant *Pseudomonas* spp. isolated from RMFs were submitted to a commercial RAPIDEC^®^ CARBA NP test: none were positive (Table 3). Screening results being consistently negative for both rectal swabs and RMFs at every sampling period, Farm B was classified as ESBL- and CP-unsuspected.

In Farm C, out of the 29 isolates of fecal origin identified to the genus-level, only one was submitted to AST (Table 2): an *E. coli* isolated from ChromID™ CARBA in May, which was pansusceptible, ESBL-/AmpC- and CIAT-negative. Out of the 97 strains of RMF origin identified to the genus-level, 46 were submitted to AST: 27 *Pseudomonas* spp., nine *P. aeruginosa*, one *P. mendocina*, four *Pseudomonas nitroreducens*, two *Aeromonas* spp., two *Aeromonas veronii* and one *Aeromonas hydrophila*. All these strains were isolated from ChromID™ CARBA and ChromID™ OXA-48. Only three *P. aeruginosa* isolated in March and May were resistant to IPM, none of them were MDR. The RAPIDEC^®^ CARBA NP tests were negative for the IPM-resistant *P. aeruginosa* strains (Table 3). Screening results being consistently negative for both rectal swabs and RMFs at every sampling period, Farm C was classified as ESBL- and CP-unsuspected.

## Discussion

Herds' statuses were assessed monthly by analyzing fecal swabs from healthy lactating dairy cows in all farms, and by analyzing RMFs in two of the three farms. When assessing the data, it should be understood the classification of isolates as being ESBL-/AmpC-/CP-positive or negative is based on their phenotype and would require further testing, especially to confirm the mobile nature of the resistance genes in ESBL-positive isolates. The farms had been selected partly because there were previous occurrences of ESBL-producing *Enterobacteriaceae* in Farms A and B, and because a CP-producing *Acinetobacter* was isolated in Farm A (9) and yet we failed to isolate any CP-producing strain and found ESBL-producing *Escherichia* only in Farm A. This contradiction with previous results might be due to a decrease in antimicrobial consumption in these farms since 2016 following the implementation of a law on veterinary antibiotics usage in France. Although we initially intended to assess the use of antimicrobials in each farm in this study, we did not include the data because they were based solely on the farmers' voluntary recordings and were not deemed objective or precise enough. Another major obstacle was the unfortunate absence of RMFs from Farm A as this hampers the comparison between rectal swabs and RMFs in the only farm where ESBL-producing *E. coli* were isolated. The overall small number of resistant strains isolated, coupled with the failure to detect CP-producing isolates preclude any statistical analyses but the implementation of a year-long monthly protocol as well as the recurrent detection of ESBL-producing MDR *Escherichia* strains in Farm A and of *Pseudomonas* strains all year-long in RMF samples in Farms B and C allow for some discussion.

In a longitudinal study in a commercial dairy farm in the UK (15), there was a significant increase in detection of ESBL-producing *E. coli* in the lactating herd in May and August, and a decrease in November. Our results are consistent with this observation since 12 out of 13 of our ESBL-producing *Escherichia* strains were isolated in March, May, June and July, i.e., during spring and summer seasons. Thermal stress could explain this surge but other stress factors have been associated with more frequently identified ESBL-producers in cattle like calving (15), changes in feeding in the last 2 weeks before sample collection (11) and more intensive farm management (13). Overall, these risk factors—except antibiotic use—should be considered as general trends and more studies are still needed to determine the ideal period of sampling to determine a herd ESBL-/AmpC-status and which sample would be most appropriate (12, 51).

To the best of our knowledge, only a handful of studies have reported the use of carbapenem agars as an initial screening step with the objective of detecting CP-producing GN bacteria in the gut of healthy cattle (11, 52) or in milk from healthy cattle (53). In our study, screening with commercial carbapenem agars led to the isolation of mainly *Pseudomonas* spp., *Ochrobactrum* spp. and *Enterobacter* spp. from fecal swabs, and of *Pseudomonas* spp., *Ochrobactrum* spp., *Aeromonas* spp., *Arcobacter* spp. and *Stenotrophomonas* spp. from RMFs. These results are consistent with the previous studies in which the main genera identified are mentioned (11, 53). In all these reports, as well as in ours, no CP production or CP-genes were evidenced despite the isolation of numerous GN strains on commercial carbapenem-supplemented agars, illustrating the overall poor specificity of these media for CP-producing strain detection when applied to cattle or raw milk compared to monitoring in human patients (54–56). At least two hypotheses can be advanced to explain this poor apparent specificity: (i) evaluation studies of commercial carbapenem-supplemented agars in humans are often performed in regions where patients are frequently colonized with CP-producing bacteria, and in populations with scarcely detected CP-producing bacteria such as cattle, screening with commercial agars are expected to exhibit lower predictive positive value (56); (ii) commensal GN bacteria isolated from cattle or raw milk on carbapenem-supplemented media are often resistant to carbapenems because of non-CP resistance mechanisms, that can be either intrinsic to certain species (36, 42) or a combination of acquired resistance mechanisms in certain strains (57, 58).

The use of McC + COX media as an initial screening step for detection of ESBL-producing *E. coli* is fairly common as it allows a presumptive diagnosis of *E. coli* colonies based on their ability to ferment lactose, conferring them a typical pink color, even if there are some strains which ferment it slowly or not at all (59–61). In some studies [e.g., (12, 51, 62)], ESBL-producing *E. coli* have been searched for only among *E. coli* which formed typical lactose-positive colonies according to the “Materials and Methods” description. Yet in our study, lactose-negative ESBL-producing *E. coli* strains were isolated at a much higher frequency than lactose-positive strains (12 vs. 1) illustrating that failure to include these strains in monitoring programs could result in serious underestimation of ESBL prevalence among *E. coli*. This microbiological difference among ESBL-producing *Escherichia* strains isolated in spring/summer and in winter might be explained by a change of the dominant genotypes in the lactating dairy cows, as described in calf cohorts in a previous longitudinal study (63).

When monitoring healthy dairy cows we isolated with our protocol a majority of non-GN organisms in fecal swabs, especially *Candida* organisms. Among these non-GN strains, most of them (74 out of 93, all farms combined) were recorded at the second reading 36–48 h after inoculation and not at the first reading 18–24 h after inoculation. Therefore, suppressing the additional 18–24 h of inoculation would greatly restrict the isolation of non-GN organisms. On the other hand, this extra-step allowed for a better differentiation between strains when monitoring RMFs and permitted a more precise isolation of morphologically different colonies. If other protocols were to keep a 36–48-h incubation period with similar media we would recommend a gram-staining before any identification step so that non-GN organisms could be rapidly excluded.

By monitoring RMFs all year-long in Farms B and C, we detected the persisting presence of Pseudomonads. This observation is consistent with previous studies (64, 65). From a public health risk point of view, none of the IPM-strains were CP-producing, which means that resistance was most likely an association of non-horizontally-transferrable resistant mechanisms as is often described in *P. aeruginosa* (58, 66, 67) and that commercial carbapenem agars were not specific enough to differentiate these strains.

This is one of the few studies reporting monitoring of resistant bacteria at the herd level by using RMFs (33, 34) and performing a longitudinal monitoring of antimicrobial resistance in dairy farms (15, 63, 68–70) rather than a single snapshot. Our results are consistent with other European studies which failed to detect CP-producing strains in cattle but showed that ESBL-phenotypes were closely associated with MDR, especially to AMP-TET-STX (46). Our results also illustrate that ESBL-status in fecal swabs can change monthly in a positive-farm, therefore the terminology of Gonggrijp et al. (51) who defined herds that tested negative as “unsuspected” seems appropriate. This variation in status could be due to low sensitivity of our method and/or to an overall low prevalence of ESBL-producing GN enteric bacteria in the dairy farms chosen in our study. Further research in other farms with different ESBL-/AmpC-/CP-statuses is needed to better assess the potential of analyzing RMF as a proxy for the dairy herd fecal contamination by resistant GN commensal bacteria. We believe monitoring CP-producing bacteria in dairy herds by using RMFs could be advantageous compared to fecal samplings, but is hindered by the lack of specificity of commercially available carbapenem-supplemented media when applied to dairy cattle.

## Data Availability Statement

The original contributions presented in the study are included in the article/Supplementary Material, further inquiries can be directed to the corresponding author/s.

## Ethics Statement

Ethical review and approval was not required for the animal study because no ethical review and approval required for this study where animal subjects were only submitted to a rectal swab. Written informed consent was obtained from the owners for the participation of their animals in this study.

## Author Contributions

SG and YM: conceptualization, validation, and supervision. VP and YM: investigation. PG, SG, and YM: resources. VP: writing—original draft preparation and visualization. PG and YM: writing—review and editing and funding acquisition. YM: project administration. All authors contributed to the article and approved the submitted version.

## Conflict of Interest

The authors declare that this study received funding from Ceva Santé Animale. The funder had the following involvement in the study: writing - review and editing. In all the other aspects of the study - design, collection, analysis, interpretation of data and decision to submit it for publication - the authors declare that the funder was not involved.

## Supplementary Material

Supplementary Figure 1 Algorithm steps followed for the characterization of the strains isolated from fecal swabs and raw milk filters on selective media. McC, MacConkey; COX, cefotaxime; DDST, double disk synergy test; CIAT, ceftazidime-imipenem antagonism test; ESBL, extended-spectrum β-lactamase; AmpC, cephalosporinase; CP, carbapenemase.

Supplementary Table 1 Sampling visit details and sample population characteristics during the year 2018.

Supplementary Table 2 Applied breakpoints for definition of “microbiological” resistance for each strain submitted to antimicrobial susceptibility testing.

Supplementary Table 3 Inhibition zone diameters for each strain submitted to antimicrobial susceptibility testing.
