# Supplementary figures and images for: Surveillance of Extended-Spectrum β-Lactamase-, Cephalosporinase- and Carbapenemase-Producing Gram-Negative Bacteria in Raw Milk Filters and Healthy Dairy Cattle in Three Farms in Île-de-France, France
Source: Front Vet Sci. 2021 Feb 10;8:633598. doi: 10.3389/fvets.2021.633598 (PMC7902890; doi:10.3389/fvets.2021.633598)

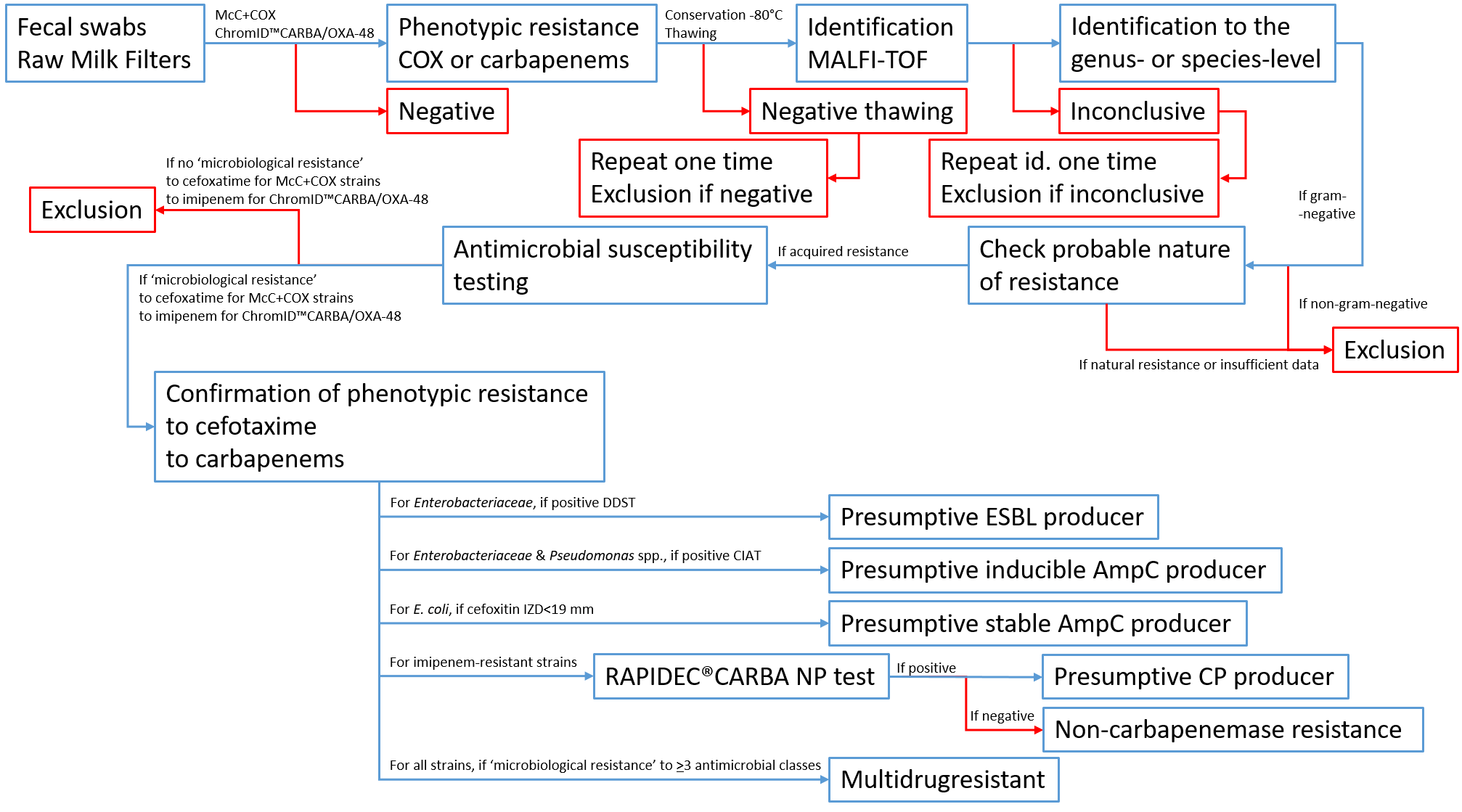

Supplement: Supplementary Figure 1 — Algorithm steps followed for the characterization of the strains isolated from fecal swabs and raw milk filters on selective media. McC, MacConkey; COX, cefotaxime; DDST, double disk synergy test; CIAT, ceftazidime-imipenem antagonism test; ESBL, extended-spectrum β-lactamase; AmpC, cephalosporinase; CP, carbapenemase. [file Image_1.PNG]
